# Melatonin for the prevention and treatment of cancer

**DOI:** 10.18632/oncotarget.16379

**Published:** 2017-03-18

**Authors:** Ya Li, Sha Li, Yue Zhou, Xiao Meng, Jiao-Jiao Zhang, Dong-Ping Xu, Hua-Bin Li

**Affiliations:** ^1^ Guangdong Provincial Key Laboratory of Food, Nutrition and Health, Department of Nutrition, School of Public Health, Sun Yat-Sen University, Guangzhou, China; ^2^ School of Chinese Medicine, Li Ka Shing Faculty of Medicine, The University of Hong Kong, Hong Kong, China; ^3^ South China Sea Bioresource Exploitation and Utilization Collaborative Innovation Center, Sun Yat-Sen University, Guangzhou, China

**Keywords:** melatonin, anticancer, mechanisms of action, receptor, apoptosis

## Abstract

The epidemiological studies have indicated a possible oncostatic property of melatonin on different types of tumors. Besides, experimental studies have documented that melatonin could exert growth inhibition on some human tumor cells *in vitro* and in animal models. The underlying mechanisms include antioxidant activity, modulation of melatonin receptors MT1 and MT2, stimulation of apoptosis, regulation of pro-survival signaling and tumor metabolism, inhibition on angiogenesis, metastasis, and induction of epigenetic alteration. Melatonin could also be utilized as adjuvant of cancer therapies, through reinforcing the therapeutic effects and reducing the side effects of chemotherapies or radiation. Melatonin could be an excellent candidate for the prevention and treatment of several cancers, such as breast cancer, prostate cancer, gastric cancer and colorectal cancer. This review summarized the anticancer efficacy of melatonin, based on the results of epidemiological,experimental and clinical studies, and special attention was paid to the mechanisms of action.

## INTRODUCTION

**Figure 1:**
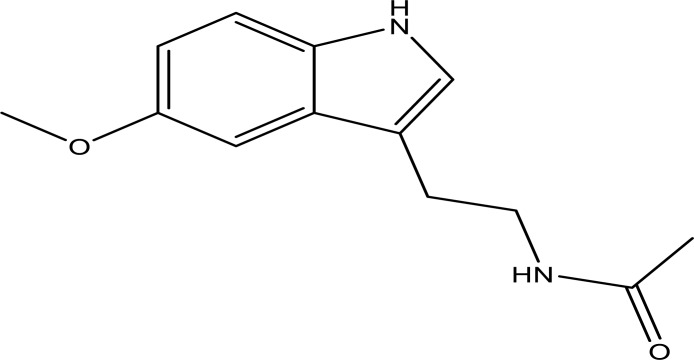
Structure of melatonin

**Figure 2:**
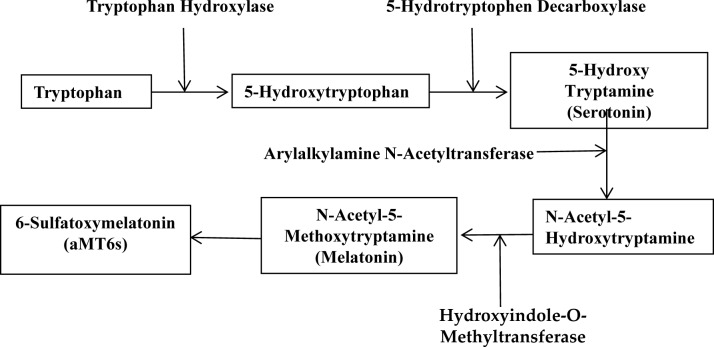
The biosynthesis and metabolism process of melatonin

Melatonin (N-acetyl-5-methoxytryptamine, Figure 1) is an indolic compound secreted primarily by the pineal gland of human and mammals in response to darkness [1]. Except for the pineal, melatonin synthesis is also found in several other organs, including the retina, gastrointestinal tract, skin, bone marrow, and lymphocytes [2]. The process of melatonin biosynthesis and metabolism is shown in Figure 2, and only the primary metabolite 6-sulphatoxymelatonin (aMT6s) is included, because it is commonly used as the maker of circadian melatonin level [3–5]. The synthesis and secretion of melatonin are regulated by the ‘master biological clock’ located in the suprachiasmatic nucleus (SCN) of the hypothalamus [6]. Although melatonin is regulated by central circadian clock, it could also modulate central circadian clock and peripheral oscillators in tissues and organs, which makes melatonin a marker of circadian rhythms [7]. The melatonin level elevates at night and decreases throughout the day. Studies have shown that increased nighttime melatonin levels in the blood could send signals to the body's cells and organs that it is nighttime and help organize target organs and organ systems into appropriate homeostatic metabolic rhythms [8]. Therefore, light at night (LAN) could disrupt the circadian rhythm and the melatonin production [9], which could contribute to the development, promotion, and progression of cancers.

According to the data reported by WHO, cancer is the leading cause of worldwide morbidity and mortality, with approximately 14 million new cases and 8.2 million cancer associated deaths in 2012 [10]. In the USA alone, it's estimated that in 2016, 1,685,210 new cancer cases and 595,690 cancer deaths could occur [11]. Nowadays, patients with cancer mainly count on clinical treatment, e.g. surgery, radiotherapy and chemotherapy. In addition, some natural products showed the potential for prevention and treatment of cancers [12–21]. Studies on cancer and anticancer therapies have attracted great attention.

In the last decades, accumulating evidence has outlined the relevance of melatonin to human physiology and pathology. Now it is well accepted that melatonin is not only a hormone, but also a cell protector [22], involved in immunomodulation, antioxidative processes, and hematopoiesis [23, 24]. Moreover, a bunch of studies have shown that melatonin has important oncostatic properties, through receptor-dependent and receptor-independent mechanisms [25]. The melatonin receptors MT1 (encoded by *MTNR1A*) and MT2 (encoded by *MTNR1B*) belong to the G-protein-coupled receptor (GPCR) group [26], and are mainly responsible for mediating the downstream effects of melatonin [27]. For incidence, they are involved in inhibition of adenyl cyclase and cyclic AMP (cAMP), leading to a reduction in uptake of linoleic acid. Melatonin-induced inhibition on linoleic acid uptake is considered as a mechanism of its antiproliferative effects [28]. The receptor-independent mechanisms are associated with antioxidant activity, regulation of apoptosis, tumor metabolism and cancer immunity, inhibition on angiogenesis and migration, and prevention of circadian disruption [25, 29, 30]. Melatonin also showed the potential to be utilized as adjuvant of cancer therapies, through reinforcing the therapeutic effects and reducing the side effects of chemotherapies or radiation [31].

The objective of the present review is to summarize recent discoveries on the oncostatic property of melatonin, classified by hormone-dependent cancers and hormone-independent cancers, and to discuss the mechanisms of action, based on the results of epidemiological research, experimental studies and clinical trials.

## EPIDEMIOLOGICAL STUDIES

Several epidemiological studies support a protective role of melatonin in cancer, yet not all the epidemiological studies are consistent (Table 1). Some studies suggested an inverse association between circadian melatonin level and breast cancer incidence. According to a dose-response analysis of observational studies, high artificial LAN exposure was related with an increased risk of breast cancer (RR = 1.17, 95% CI: 1.11-1.23), and risk of breast cancer was reduced by 14% with an increase of 15 ng/mg creatinine in urinary aMT6s (RR = 0.86, 95% CI: 0.78-0.95), with a linear dose-response trend (P-trend = 0.003) [32]. In addition, a case-control study found that female subjects with serum melatonin levels ≤ 39.5 pg/mL had a significantly higher risk of breast cancer incidence (about 15 folds) compared with subjects with levels > 39.5 pg/mL (OR = 14.24; 95% CI = 4.32-46.90). Meanwhile, the GG genotype of the *MTNR1b* (encoding melatonin receptor MT2) gene rs#10830963 polymorphism significantly elevated the risk of breast cancer by about 21 times more than the CC genotype (OR = 20.67; 95% CI = 4.77-99.33) [33]. Besides, a meta-analysis including 5 prospective case-control studies reported an inverse relationship between breast cancer risk and the highest levels of urinary aMT6s [34]. Another study evaluated the association between breast cancer risk and common single nucleotide polymorphismsin the *MTNR1a, MTNR1b*, and *AANAT* (encoding arylalkylamine N-acetyltransferase) genes among 2,073 cases and 2,083 controls, and reported that common genetic variation in the *MTNR1a* and *MTNR1b* genes might contribute to breast cancer susceptibility, and the associations might vary with menopausal status [27]. A nested case-control study reported that a higher urinary aMT6s level was significantly associated with a lower risk of breast cancer (OR = 0.62; 95% CI, 0.41-0.95; P(trend) = 0.004) [35]. However, 4 case-control studies suggested there was no evidence that melatonin level was associated with breast cancer risk. A prospective nested case-control study among British women pointed out that no statistically significant differences in urinary aMT6s level between women with breast cancer and healthy women were observed, regardless of menopausal status [36]. Besides, a case-control study nested in the Women's Health Initiative Observational Cohort reported there was no evidence that higher urinary levels of melatonin were inversely related with breast cancer risk in postmenopausal women [37]. Results from another case-control study nested within the Nurses' Health Study II cohort also did not support an overall association between urinary melatonin levels and breast cancer risk [38]. Likewise, no significant association was found between aMT6s level and breast cancer risk (either overall or by menopausal status) in a case-control study nested in the Guernsey III Study [39].

**Table 1:** Epidemiological studies on melatonin level and cancer risks

Cancer	Study Type	Association	Ref.
breast cancer	dose-response analysis of observational studies	RR= 0.86, 95% CI= 0.78-0.95	[32]
breast cancer	case-control study	OR=14.24, 95% CI= 4.32-46.90	[33]
breast cancer	meta-analysis	fixed effects model: 95% CI = 0.71-0.95, *p* = 0.01; random effects model: 95% CI = 0.68-0.99, p = 0.04	[34]
breast cancer	case-control study	premenopausal women: OR = 1.57, 95% CI = 1.07–2.31, *p* = 0.020; postmenopausal women: OR = 0.58, 95% CI = 0.36–0.95, *p* = 0.030	[27]
breast cancer	nested case-control study	OR = 0.62; 95% CI, 0.41-0.95; *p* = 0.004	[35]
breast cancer	nested case-control study	no significant association	[36]
breast cancer	case-control study	no significant association	[37]
breast cancer	case-control study	no significant association	[38]
breast cancer	case-control study	no significant association	[39]
prostate cancer	case-cohort study	HR = 4.04, 95% CI: 1.26-12.98	[40]
prostate cancer	case-control study	prostate cancer: aOR= 0.59, 95% CI: 0.35-0.99 advanced stage prostate: aOR= 0.49, 95% CI= 0.26-0.89	[41]
ovarian cancer	retrospective study	*p* < 0.05	[42]
solid tumors	meta-analysis	1-year survival rate: RR = 1.90; 95% CI= 1.28-2.83	[43]
solid tumors	meta-analysis	RR = 0.60; 95% CI= 0.54-0.67	[44]
ovarian cancer	case-control study	no significant association	[45]

As for cancers other than breast cancer, a case-cohort study reported that men with first morning urinary aMT6s levels below the median possessed a fourfold higher risk of prostate cancer compared with men with levels above the median (HR: 4.04; 95% CI: 1.26-12.98) [40]. In addition, a case-control study pointed out that patients with high melatonin-sulfate levels or a high melatonin-sulfate/cortisol ratio were less likely to have prostate cancer (adjusted OR (aOR) = 0.59, 95% CI: 0.35-0.99; aOR = 0.46, 95% CI: 0.27-0.77) or advanced stage prostate (aOR = 0.49, 95% CI = 0.26-0.89; aOR = 0.33, 95% CI = 0.17-0.62) [41]. A retrospective study found that the serum melatonin levels in women with ovarian cancer were significantly lower compared with control subjects (*p* < 0.05), indicating that reduction in circulating melatonin level might contribute to the pathogenesis of ovarian cancer [42]. Besides, according to a meta-analysis of RCTs, melatonin significantly improved the complete and partial remission (16.5 *vs*. 32.6%; RR = 1.95, 95% CI: 1.49-2.54; *p* < 0.00001), 1-year survival rate (28.4 vs. 52.2%; RR = 1.90; 95% CI: 1.28-2.83; *p* = 0.001) for solid tumors, and markedly decreased side effects induced by radiochemotherapy, including neurotoxicity, thrombocytopenia, and fatigue. Meanwhile, effects were accordant across different types of cancers [43]. Similarly, another meta-analysis summarizing 21 clinical trials, which all dealt with solid tumors, revealed that melatonin as an adjuvant cancer care with chemotherapy decreased 1-year mortality (RR = 0.60; 95% CI: 0.54-0.67), and reduced chemotherapy-induced symptoms such as asthenia, leucopenia, nausea, vomiting, and hypotension [44]. However, a nested case-control study showed that no obvious association between urinary melatonin level and ovarian cancer risk was observed [45].

It should be noted that in the existing epidemiological studies, the methods of melatonin assessment are not uniformed, since melatonin concentrations were measured in different samples, such as urine, plasma or serum. Moreover, the melatonin concentration in human body changes with circadian rhythm, however, it has not been determined which sample collection time could best reflect the biological effects of melatonin. These differences might partially result in the inconsistence of epidemiological studies. In this case, the laboratory study in cell culture or on animal models might be more clear and direct to assess the anticancer effect of melatonin and investigate the possible mechanisms involved in this process.

## EXPERIMENTAL STUDIES

Accumulating evidence from experimental studies has supported the anticancer properties of melatonin. Given the vast number of studies, publications in the PubMed and Web of Science databases were searched and relevant peer-reviewed articles published in English within 5 years were identified.

### Hormone-dependent cancer

### Breast cancer

Breast cancer is one of the most common cancers in women, and one of the leading causes of death for women aged 40 to 55 years [46] . Research concerning the melatonin's effects on breast cancer is the largest, possibly due to that melatonin has shown to modulate several aspects of endocrine physiology. Early studies reported that melatonin produced antiproliferative effect in breast cancer cells *in vitro* [47], and inhibited the growth of mammary tumors in rats [48, 49]. Afterwards, different mechanisms of melatonin's anticancer effects were identified successively, such as inducing apoptosis [50], antiestrogenic effect through ERα-signaling pathway and inhibiting aromatase activity [51–53], modulation of melatonin receptors [54], and inhibition on invasion [55] and angiogenesis [56].

### *In vitro* studies

Melatonin presented an anti-metastatic effect on CMT-U229 and MCF-7 breast cancer cell lines, through inhibiting the viability and invasiveness of breast cancer mammospheres and also mediating expression of epithelial mesenchymal transition (EMT) related proteins [57]. The anti-invasive effect of melatonin on breast cancer was also through down-regulation of the p38 pathway and suppression of MMP-2 and -9 expression and activity [58]. Melatonin also decreased proliferation and viability and induced apoptosis in neoplastic mammary cells, with better efficacy in ER-positive tumors which presented a high expression of melatonin receptor MT1 [59]. In addition, melatonin exhibited an antiproliferative activity and stimulated apoptosis in breast cancer cell MDA-MB-361, through a simultaneous modulation of the COX-2/PGE2, p300/NF-κB, and PI3K/Akt/signaling and activation of the Apaf-1/caspase-dependent apoptotic pathway [60].

It has been demonstrated that melatonin might control tumor growth in advanced cancer patients, which was at least in part through functioning as a natural anti-angiogenic molecule as evidenced by decreased blood VEGF levels [61]. For breast cancer, 1 nM melatonin reduced the viability of hypoxic MCF-7 and MDA-MB-231 human breast cancer cells and decreased both gene and protein expression of HIF-1α and VEGF-A (*p* < 0 .05), indicating an anti-angiogenesis effect [56]. Additionally, Alvarez-Garcia *et al*. reported the anti-angiogenesis effect of melatonin by downregulation of VEGF expression in human breast cancer cells, which decreased the levels of VEGF around endothelial cell [62]. Another study also observed that melatonin was effective in controlling tumor angiogenesis [63].

Melatonin could inhibit aromatase activity in breast cancer cells. Melatonin of 20 nM generated an anti-aromatase effect as potent as 20 nM letrozole, which is a selective estrogen enzyme modulator and clinically used anti-aromatase drug in breast cancer treatment [64]. A possible mechanism of melatonin's anti-aromatase effect was through inhibition on COX activity and expression [65]. Likewise, melatonin treatment exerted significant inhibition on *CYP19A1* gene transcription and aromatase activity on breast adipose fibroblasts and breast cancer-associated fibroblasts [66]. Besides, melatonin could interfere with the desmoplastic reaction in breast cancer, *via* down-regulating the expression of anti-adipogenic cytokines, which could promote the differentiation of fibroblasts and produce an anti-aromatase action, thereby decreasing the number of estrogen-producing cells proximal to malignant cells [67].

A group reported that melatonin could induce differential expression of miRNA and miRNA-related genes in human breast cancer cells, indicating an oncostatic effect [68, 69]. Furthermore, they reported that melatonin treatment expressed an anticancer effect through influencing DNA methylation patterns, by down-regulating oncogenic genes (*EGR3* and *POU4F2/Brn-3b*) and up-regulating the tumor suppressor gene (GPC3) [70]. Besides, long-term treatment of melatonin could suppress proliferation and migration of breast cancer cells partially through downregulation of miR-24, which was regarded as a potential diagnostic tumor marker [71]. Treating MT1-transfected MCF-7 cells with melatonin could lead to a significant amplified growth-inhibition, while MT1-transfected MDA-MB-231 cells showed no response to melatonin, indicating the mediation role of MT1 receptor in melatonin's oncostatic activity [72]. Additionally, among 3 ER-negative breast cancer cell lines, only the proliferation of SK-BR-3 cells was inhibited by melatonin, despite that MT1 receptor was expressed in all three cell lines [73].

The synergetic effect of melatonin with other anticancer drugs or radiotherapy is also noteworthy. Melatonin enhanced the effects of doxorubicin by activating transient receptor potential vanilloid 1 (TRPV1) and apoptosis as well as inducing MCF-7 cell death [74]. In addition, melatonin enhanced the apoptotic cell death induced by arsenic trioxide *via* ROS generation, upregulation of Redd1 expression, and activation of the p38/JNK pathways in human breast cancer cells [75]. Besides, melatonin (3 mM) combined with puromycin (1 μM) exerted synergistically inhibitory effect on MDA-MB 231 cells, through attenuating the expression of 45S pre-rRNA and upstream binding factor, and downregulating upstream binding factor, XPO1 and IPO7, procaspase 3, and Bcl-xL [76]. Furthermore, combined treatment of melatonin with all-trans retinoic acid and somatostatin enhanced the inhibitory effect on viability and growth of MCF-7 cells [77]. In addition, melatonin combined with vitamin D_3_ synergistically inhibited proliferation with an almost complete cell growth arrest in MCF-7 cells [78]. Melatonin could also enhance the radiosensitivity of cancer cells. Pretreatment with melatonin on MCF-7 breast cancer cells 1 week before radiation could sensitize cancer cells to the ionizing effects of radiation, through inhibiting proliferation, promoting cell cycle arrest and inhibiting proteins involved in double-strand DNA break repair [79]. Another study suggested the enhancing effect of melatonin on the radiosensitivity of human breast cancer cells might be associated with the increase of p53 expression [80].

#### *In vivo* studies

A study using female athymic nude mice reported that melatonin could control metastatic breast cancer through decreasing ROCK-1 expression [81]. In addition, melatonin treatment on nude mice with breast cancer xenografts reduced tumor size and cell proliferation compared to control animals, along with a decrease in expression of VEGF receptor 2 and micro-vessel density, indicating the inhibition of angiogenesis [82]. LAN is a problem co-distributed with breast cancer prevalence worldwide [83]. A study documented LAN's positive effect on breast tumors growth rate, and melatonin could reverse the effect *via* global DNA methylation [83]. Moreover, another study elucidated the association between LAN-induced circadian disruption and the increased breast cancer risk: LAN disrupted the circadian rhythm of glycogen synthase kinase 3β (GSK3β) through perturbation of the nocturnal surge of melatonin, and melatonin activated GSK3β *via* inhibiting the serine-threonine kinase Akt phosphorylation and inducing β-catenin degradation and inhibiting EMT [84].

Several studies reported the synergetic effect of melatonin with other anticancer agents on breast cancer. Melatonin co-administration significantly augmented the pro-differentiating, antiproliferative and immunomodulatory activities presented by *Lactobacillus plantarum* LS/07 and inulin [85]. In addition, combined treatment of melatonin with *Propionibacterium acnes* on Balb/C mice transplanted with EMT6/P cell line could inhibit metastasis of cancer cells to other organs, induce apoptosis, reduce angiogenesis, and stimulate strong Th1-type cytokine antitumor immune response [86]. Another study reported melatonin's enhancing effect on the sensitivity of breast tumor to adriamycin [87]. Furthermore, melatonin could potentiate the anti-tumor effect of pravastatin in rats with mammary gland carcinoma, as shown by decreased tumor frequency by 69% and lengthened tumor latency by nine days compared with control animals [88]. Additionally, doxorubicin resistance and tamoxifen resistance in breast cancer were caused by LAN-induced disruption of circadian melatonin signal, and melatonin could inhibit tumor metabolism and circadian-regulated kinase to reestablish the sensitivity of breast tumors to doxorubicin [89] and tamoxifen [90].

Collectively, melatonin has exhibited inhibitory effect on both ER-positive and ER-negative breast cancers. Melatonin's anti-breast cancer effect was not only mediated by its interaction with both the estrogen receptors and the melatonin receptors, but also through activating various receptor-independent and estrogen-independent signaling pathways. Given the wide spectrum of melatonin's action on breast cancer, coupled with its low toxicity, it could be considered as a potential therapeutic choice for prevention and treatment of breast cancer.

#### Prostate cancer

Prostate cancer is the second most frequently occurred cancer and the fifth leading cause of cancer mortality in men [91]. It was found that melatonin at pharmacological concentrations could inhibit cell growth of both androgen-dependent and androgen-independent prostate cancer [92], through various mechanisms.

#### *In vitro* studies

Melatonin significantly suppressed the expression of angiogenesis-related proteins HIF-1α, HIF-2α and VEGF at mRNA level of PC-3 cells under hypoxia, and upregulation of miRNA3195 and miRNA374b could mediate this anti-angiogenic property of melatonin [93]. Furthermore, melatonin presented anti-proliferative effects on prostate cancer cell lines, LNCaP and 22Rv1, and the mechanism might involve inactivation of NF-κB, via melatonin MT1 receptor-induced dual activation of (Gα_s_)/protein kinase A (PKA) and (Gα_q_)/protein kinase C (PKC), causing transcriptional upregulation of p27^Kip1^. The mechanism also involved downregulation of activated AR signaling *via* PKC stimulation [94, 95]. Another study documented that melatonin suppressed HIF-1α accumulation *via* inactivating sphingosine kinase 1 pathway and scavenging free radicals in hypoxic PC-3 cells, thus melatonin could act as a potent anti-cancer supplement for prostate cancer therapy [96]. Sirt1 (sirtuin 1) is a NAD^+^-dependent histone deacetylase and overexpressed in prostate cancer cells [97]. Melatonin significantly suppressed Sirt1 activity *in vitro* in multiple human prostate cancer cell lines, accompanied by a significant reduction in the proliferative potential of PCa cells [98]. In addition, melatonin could cause phenotypic changes, mainly neuroendocrine differentiation, thereby sensitizing human prostate cancer cells to apoptosis induced by cytokines, such as TNF-α or TRAIL [99]. Overexpression of Period 2 (Per2) gene could lead to a significant decrease of PCa cell growth and viability, and melatonin treatment could inhibit proliferation of prostate cancer cells by resynchronizing dysregulated circadian rhythm circuitry through upregulating Per2 and Clock genes and downregulating Bmal1 [100].

#### *In vivo* studies

Daytime blue light could increase nocturnal melatonin and then enhance the inhibition on human prostate cancer growth on male nude rats, as shown by decreased tumor growth rates, tumor cAMP levels, aerobic glycolysis (Warburg effect), uptake-metabolism of linoleic acid, and growth signaling activities [101]. In another study, melatonin in LNCaP human prostate cancer cells xenografted mice inhibited the xenograft growth rate by exerting an anti-angiogenesis effect by reducing xenograft microvessel density and expression of Ki67, and elevating expression of HIF-1α and phosphorylation of Akt. Melatonin also restored the redox imbalance by promoting expression of Nrf2 [102]. Furthermore, human volunteer nighttime-collected, melatonin-rich blood dampened signal transduction, metabolic and growth activity in tissue-isolated PC-3 cancer xenografts, *via* a melatonin MT1 receptor-mediated mechanism. On the contrary, blood collected from human subjects exposed to LAN exerted an exactly opposite effect via suppression of the nocturnal circadian melatonin signal [103]. Besides, oral administration of melatonin significantly inhibited prostate cancer tumorigenesis as characterized by reduction in prostate and genitourinary weight, serum IGF-1/IGFBP3 ratio, and mRNA and protein levels of PCNA and Ki-67, which were accompanied with a significant reduction in Sirt1 [98].

Collectively, these scientific literatures support the potential application of melatonin in the prevention and treatment of prostate cancer. Especially, melatonin could exert antiproliferative activity on androgen-independent prostate cancer cells (e.g. PC-3 cells), which makes melatonin a clinical choice to postpone the relapse of hormone-refractory or castration-resistant prostate cancer in combination with androgen deprivation therapy.

### Ovarian cancer

Ovarian cancer is one of the leading cause of death among women with genital tract disorders [104]. Although various surgical techniques and chemotherapies have been applied to the treatment of ovarian carcinoma, the prognosis remains poor [105]. In recent years, a few studies have reported the anticancer effect of melatonin on ovarian cancer.

#### *In vitro* studies

Melatonin induced an accumulation of OVCAR-429 and PA-1 ovarian cancer cells in the G_1_ phase *via* downregulation of CDK 2 and 4 [106]. Besides, although melatonin alone showed no significant cytotoxicity against SK-OV-3 human ovarian cancer cells, melatonin could synergistically enhance cisplatin-induced apoptosis. The pro-apoptotic effect was through inactivating caspase-3 and promoting cisplatin-mediated inhibition of extracellular signal regulated kinase (ERK), 90-kDa ribosomal S6 kinase (p90RSK) and heat shock protein 27 (HSP27) phosphorylation [107].

#### *In vivo* studies

A group studied the oncostatic effect of melatonin on ovarian cancer using an ethanol-preferring rat model, in which the left ovary was inoculated with ovarian tumor and right ovary was used as the sham-surgery control [108–110]. They found that melatonin could reduce ovarian tumor masses and decrease the incidence of adenocarcinoma in rats [108]. Later, they investigated the apoptosis-promoting effect of melatonin on ovarian cancer. Results showed that absolute and relative tumor masses were significantly reduced after melatonin therapy, regardless of ethanol consumption. Melatonin therapy promoted apoptosis as characterized by upregulation of p53, BAX, and cleaved caspase-3, as well as enhancement of DNA fragmentation [109]. Furthermore, the group found that melatonin attenuated the TLR4-induced MyD88- and TRIF-dependent signaling pathways in rats with ovarian cancer [110]. In addition, epidermal growth factor receptors 2 (Her-2) and 4 (Her-4) were closely related with the progression and metastasis of ovarian cancer [111]. A study found that melatonin could attenuate the Her-2-signaling pathway in the ethanol-preferring rat model, by significantly suppressing the expression of Her-2, p38 MAPK, and p-Akt [112].

Collectively, melatonin has shown anticancer effect on ovarian cancer, and the underlying mechanisms include inducing apoptosis and cell cycle arrest, and immunoregulation (toll-like receptors).

### Cervical cancer

Cervical cancer is the second leading cause of female tumor worldwide, and its incidence in developing countries is much higher than that in developed countries [113]. The anticancer effect of melatonin on cervical cancer has been reported in a few studies.

#### *In vitro* studies

Melatonin decreased HeLa cell viability, and significantly enhanced the cytotoxic effect of 3 chemotherapeutic agents (cisplatin, 5-fluorouracil, and doxorubicin), as shown by increased caspase-3 activation. Especially, co-treatment of melatonin and cisplatin significantly elevated the ratio of cells entering mitochondrial apoptosis though ROS overproduction, and markedly enlarging DNA fragmentation compared to cisplatin treatment alone [114].

#### *In vivo* studies

Melatonin inhibited growth of HeLa cervical cancer xenografts perfused *in situ* in nude rats, via inhibiting aerobic glycolysis (Warburg effect) and fatty acid metabolic signaling [115]. Furthermore, melatonin suppressed HeLa cervical adenocarcinoma metabolism and proliferation through inhibition of linoleic acid transport and 13-hydroxyoctadecadienoic acid production via a receptor-mediated signal transduction [116].

Melatonin could reduce cervical cancer cell viability *in vitro*, and suppress cervical adenocarcinoma metabolism *in vivo*. More studies are required to fully explain the oncostatic effect of melatonin on cervical cancer and support the clinical application of melatonin.

### Endometrial cancer

Endometrial cancer, like breast cancer, is an estrogen-dependent neoplasm, and its incidence is rapidly increasing worldwide [117, 118]. The effect of melatonin on endometrial cancer was only reported in few studies.

#### *In vivo* studies

Visceral obesity is a risk factor of endometrial cancer, as it is associated with chronic inflammatory process [119]. Ciortea *et al*. reported that, compared with estrogen replacement treatment, the combinational treatment of melatonin and estrogen in ovariectomized rats was associated with lower body weight, less intra-retroperitoneal fat, reduction in endometrial proliferation, and less appearance of cellular atypia. These results indicated that melatonin supplementation might be used in the prophylaxis of endometrial cancer in menopause women [119].

### Hormone-independent cancers

#### Oral cancer

Oral cancer is a common type of human head and neck cancers, and most of the cases involve oral squamous cell carcinoma [120]. In several *in vitro* studies, melatonin has shown noteworthy effect on oral cancer.

In a study, melatonin presented an anti-metastatic effect on oral cancer cell lines (HSC-3 and OECM-1), through attenuation of MMP-9 expression and activity, which was mediated by decreasing histone acetylation [121]. Besides, melatonin could decrease the cell viabilities of SCC9 and SCC25 cell lines (both squamous cell carcinoma of the tongue), and exert an inhibitory effect on the expression of pro-metastatic gene, *ROCK-1*, and pro-angiogenic genes, *HIF-1α* and *VEGF* in SCC9 cell line [122].

Collectively, melatonin has shown inhibitory effect on some oral cancer cells, and the underlying mechanisms mainly involved inhibition on metastasis and angiogenesis.

#### Liver cancer

Liver cancer is the second most common cause of cancer death globally, and hepatocellular carcinoma (HCC) is the major kind of liver cancer (70%-80%), which is one of the most frequent cancers with the highest incidence in developing countries [123, 124]. Surgery remains the most effective treatment for patients with HCC, but it is only suitable to limited cases, thus finding effective chemotherapeutic drug is required [125]. The effects of melatonin on liver cancer have been reported in several studies.

#### *In vitro* studies

A study revealed the underlying mechanism of melatonin's anti-invasive activity in HepG2 liver cancer cells, which was through suppressing MMP-9 gelatinase activity, downregulating MMP-9 gene expression, upregulation of tissue inhibitor of metalloproteinases (TIMP)-1, and depressing NF-κB translocation and transcriptional activity [126]. Furthermore, melatonin also showed anti-angiogenic effects on HepG2 liver cancer cells through interfering with the transcriptional activation of VEGF, reducing Hif1α protein expression and STAT3 activity [127]. Additionally, it's well established that inhibitor of apoptosis proteins (IAPs) play crucial parts in apoptosis resistance, and one study documented that melatonin could overcome apoptosis resistance in human hepatocellular carcinoma by suppressing survivin and XIAP (both are members of IAPs) *via* the COX-2/PI3K/AKT pathway [128]. Furthermore, melatonin exerted pro-apoptotic effect *via* upregulating expression of Bcl-2-interacting mediator (Bim) by FoxO3a on HepG2 hepatocarcinoma cells, through activation of the transcription factor FoxO3a and increased its nuclear localization. Meanwhile, the induced apoptosis was not observed in primary human hepatocytes (used as control) [129]. Another study documented that melatonin reduced cell viability and inhibited the proliferation of HepG2 cells, which was modulated by the MT1 membrane receptor. Meanwhile, the decrease of cAMP level and increase of ERK activation induced by melatonin were also responsible for the inhibitory effect [130]. Endoplasmic reticulum (ER) stress-mediated cell apoptosis is involved in cancer development and progression. Another study revealed that melatonin could sensitize human hepatoma cells to ER stress-induced apoptosis through downregulating the COX-2 expression and the Bcl-2/Bax ratio, and elevating the levels of C/EBP homologous protein (CHOP) [131].

#### *In vivo* studies

In a study, melatonin reversed the alteration caused by N-nitrosodiethylamine-induced liver tumor in liver marker enzymes (ALT, AST), antioxidant levels, and circadian clock disturbance in mice [132]. Another study suggested that melatonin attenuated hepatocarcinogenesis induced by diethylnitrosamine in rats, by activating ER stress and inducing apoptosis [133].

Collectively, melatonin could inhibit the process of hepatocarcinogenesis mainly through the pro-apoptotic (via modulation of COX-2/PI3K/AKT pathway, Bcl-2/Bax ratio, activation of ER stress), anti-angiogenesis and anti-invasive effects.

### Renal cancer

Renal cancer is highly aggressive and the third most common urologic cancer, which accounts for approximately 3% of adult cancers with a male predominance (sex ratio 3/1) [134]. The research on melatonin's anticancer effect on renal cancer is summarized.

#### *In vitro* studies

A recent study systematically investigated the anti-metastatic effect of melatonin on renal cell carcinoma (RCC) [135]. Melatonin inhibited MMP-9 transactivation and tumor metastasis though inhibiting Akt-MAPKs pathway and NF-κB DNA-binding activity. Moreover, clinical sample analysis found a higher survival rate in MTNR1A^high^/MMP-9^low^ patients than in MTNR1A^low^/MMP-9^high^ patients [135]. Melatonin also induced apoptosis by upregulating the expression of Bim in renal cancer Caki cells, at both transcriptional level and post-translational level [136]. Furthermore, co-treatment of melatonin (1 mM) and thapsigargin (50 nM) induced more apoptosis in human renal cancer cells than treatment with thapsigargin alone, which was through ROS-mediated upregulation of CCAAT-enhancer-binding protein homologous protein [137]. Co-treatment of melatonin and kahweol induced apoptosis, stimulated DEVDase activity (could reflect caspase-3 activity), and DNA fragmentation of Caki renal cancer cells. The mechanism underlying was elucidated as inducing upregulation of p53-upregulated modulator of apoptosis [138].

Collectively, inducing apoptosis and inhibiting metastasis are the main effects of melatonin on renal cancer cells. Moreover, concomitant melatonin ministration with other therapies might be an effective clinical choice for patients with renal cancer, given that melatonin showed enhancement effect on other anticancer agents.

### Lung cancer

Lung cancer is a leading cause of cancer-related death. For instance, lung cancer is the second most frequent type of cancer in males with approximately 17,330 new cases identified in 2016 in Brazil [139]. Non-small-cell lung cancer (NSCLC) is a major form of lung cancer [140], and the published literature have suggested that the disruption of melatonin rhythm could increase the NSCLC incidence [141]. In several studies, melatonin has been reported to be a potential therapeutic strategy for lung cancer, mainly because melatonin showed to enhance the effects of radiotherapy and some anticancer drugs.

#### *In vitro* studies

In a study, it was found that in cell cycle and apoptosis regulator 2 (CCAR2)-deficient cancer cells, melatonin augmented the apoptosis induced by genotoxic stress caused by UV irradiation. The results indicated that melatonin could be a potential supplement to classical antitumor drugs in therapies against CCAR2-deficient cancers [142]. In addition, melatonin significantly suppressed the migration and viability of A549 cells, which might be through downregulation of the expression of osteopontin, myosin light chain kinase, phosphorylation of myosin light chain, and upregulation of the expression of occludin involving JNK/MAPK pathway [143]. A study showed melatonin effectively increased the berberine-induced inhibitions of cell proliferation, migration and apoptosis. The enhancing effect was possibly through activation of caspase/Cyto C and inhibition of AP-2β/hTERT, NF-κB/COX-2 and Akt/ERK signaling pathways [140]. In addition, the use of EGFR tyrosine kinase inhibitors (TKIs) to treat advanced NSCLC patients has become a standard of care, but NSCLC patients with somatic EGFR mutations, particularly T790M, showed drug resistance to TKIs. Treatment of melatonin with gefitinib (a type of TKI) effectively decreased the viability of H1975 cells containing the T790M somatic mutation, downregulated EGFR phosphorylation, and induced apoptosis compared to treatment with gefitinib or melatonin alone [144].

Judging from the available evidence, melatonin's effect was more significant when it was used as an adjuvant therapy than being used alone for lung cancer. The enhancement of melatonin on the therapeutic effects of gefitinib, berberine and doxorubicin demonstrated its beneficial role in prevention and treatment for lung cancer.

### Gastric cancer

Gastric cancer is one of the most common forms of cancer worldwide, and causes a mortality rate ranking second among malignant tumors worldwide [145]. It was estimated that there were 951,600 new cases and 723,100 deaths from gastric cancer in 2012 worldwide [146]. Melatonin has been reported to inhibit gastric cancer through various mechanisms in several studies.

#### *In vitro* studies

In a study, melatonin inhibited HIF-1α accumulation and endogenous VEGF generation through inhibition of RZR/RORγ in hypoxic SGC-7901 cells, thus inhibiting the proliferation of gastric cancer cells [147]. Besides, melatonin inhibited angiogenesis in SGC-7901 gastric cancer cell line as shown by decreased expression of VEGF mRNA and protein and suppressed expression of nuclear receptor RZR/RORγ mRNA and protein [148]. In addition, melatonin was able to inhibit cell viability, clone formation, cell migration and invasion, and induce apoptosis of AGS gastric cancer cell line, through the activation of JNK and P38 MAPK and the suppression of NF-κB. Moreover, melatonin significantly potentiated the anti-tumor effect of cisplatin with low systemic toxicity [149]. Melatonin's inhibitory effects on cell proliferation, colony formation and migration efficiency, and pro-apoptotic effect were also shown on gastric adenocarcinoma cell line SGC7901 [150]. In addition, melatonin inhibited murine foregastric carcinoma (MFC) cell growth in dose- and time-dependent manners, through increase of p21 and Bax and decrease of Bcl-2, which was mediated by membranous melatonin receptors [151]. In another study, melatonin promoted apoptosis dose-dependently and induced cell cycle arrest at the G_2_/M phase in MFC cells, and the mechanism was associated with downregulation of CD4^+^ and CD25^+^ regulatory T cells and its Forkhead box p3 (Foxp3) expression in the tumor tissue [152]. Additionally, gastric cancer cells SGC7901 cultured with melatonin showed more differentiated morphologic phenotype as compared with untreated cells, accompanied with upregulation of gene expression of endocan and downregulation of alkaline phosphatase and lactate dehydrogenase activities, two enzymes that promote de-differentiation in gastric tissue [153].

#### *In vivo* studies

A study found melatonin-induced C/EBPβ and NF-κB suppression could impede both gastric tumor growth and peritoneal dissemination *via* inhibiting EMT and inducing ER stress [154]. In addition, melatonin reduced the tumor volume and weight of tumor in gastric tumor-bearing nude mice, and inhibited proliferation and angiogenesis through suppressing the expression of RZR/RORγ, Sentrin-specific protease 1 (SENP1), HIF-1α, and VEGF [148]. In another study, mice were inoculated with MFC cells and treated with different doses of melatonin (0, 25, 50, and 100 mg/kg, i.p.). Results showed that tumor tissue was reduced and Tregs numbers and Foxp3 expression in the tumor tissue were inhibited by melatonin treatment [152].

In general, melatonin has shown noteworthy inhibitory effect on the growth of gastric cancer cells. The underlying mechanisms mainly included inhibiting angiogenesis, promoting apoptosis and immunoregulation effect.

### Pancreatic cancer

Pancreatic cancer is a highly lethal disease with a relatively low 5-year survival rate [155, 156]. It responds poorly to radiotherapy and chemotherapy because the tumor cells are resistant to apoptosis [157].

#### *In vitro* studies

In a study, 1 mM concentration of melatonin exhibited high inhibitory effect on cellular proliferation of pancreatic carcinoma cells (PANC-1), along with a significantly decrease in VEGF [158]. Besides, melatonin reduced viability of pancreatic tumor AR42J cells through inducing changes of mitochondrial activity and activating caspase-3 [159]. Furthermore, melatonin alone or combined with gemicitabine exhibited growth inhibition on pancreatic cancer cell line SW-1990. The mechanism was through downregulation of Bcl-2 expression and upregulation of Bax expression [160]. Furthermore, melatonin enhanced cytotoxicity and apoptosis induced by 3 chemotherapeutic agents (5-fluorouracil, cisplatin and doxorubicin) in AR42J pancreatic cancer cells *in vitro* [161]. The enhancing effect was characterized as elevated intracellular ROS production, enhanced mitochondrial membrane depolarization, and increased the population of apoptotic cells.

#### *In vivo* studies

In a study, melatonin alone or combined with gemicitabine inhibited the growth of transplanted tumors in nude mice, through its pro-apoptotic and pro-necrotic effect *via* downregulation of Bcl-2 expression and upregulation of Bax expression [160]. Besides, melatonin could improve antitumor activity of capecitabine in pancreatic cancer [162]. Results showed that in group of treatment with capecitabine and melatonin alone, pancreatic adenocarcinoma induced by N-nitrosobis(2-oxopropyl)amine was observed in 66% and 33% of the animals, respectively. However, in the group treated with combination of capecitabine and melatonin, only 10% of animals showed pancreatic adenocarcinoma.

In general, melatonin has shown inhibition on the growth of some pancreatic cancer cells. Furthermore, melatonin could enhance the efficacy of other anticancer agents on pancreatic cancer, especially gemcitabine, which is currently the standard chemotherapy for pancreatic cancer.

### Colorectal cancer

Colorectal cancer is one of the major causes responsible for cancer death worldwide [163], and in several studies, melatonin has shown anticancer potency for various colorectal cancers.

#### *In vitro* studies

A study showed that melatonin increased ROS levels and decreased cellular viability of HCT 116 human colorectal carcinoma cells [164]. Melatonin's oncostatic effect was also associated with its antioxidative and anti-inflammatory activities, counteracting the oxidative status and inhibiting the nitric oxide production in cultured colon cancer cells [165]. In another study, cell proliferation was suppressed significantly and apoptosis was induced by melatonin on colorectal cancer LoVo cell at pharmacological concentrations in a dose-dependent manner [166]. The mechanism underlying was explained as through histone deacetylase 4 (HDAC4) nuclear import and subsequent H3 deacetylation via the inactivation of Ca^2+^/calmodulin-dependent protein kinase IIα (CaMKII). Endothelin-1 (ET-1) is a peptide, which acts as a protector of carcinoma cells from apoptosis and promoter of angiogenesis [167]. A study showed that melatonin might inhibit tumor growth and progression of colon carcinoma *via* repressing the activation of ET-1 [168]. Another study investigated the ultrastructural aspect of melatonin cytotoxicity on Caco-2 human colon adenocarcinoma cell line. Results showed that ultrastructurally, Caco-2 cells showed morphological changes in melatonin treatments at 1.56 and 0.78 μg/mL with characteristics of cell degeneration as shown by the absence of microvilli, mitochondrial degeneration, presence of numerous vacuoles, and nuclear fragmentation, indicating that melatonin could promote cytotoxicity in Caco-2 cells [169]. Furthermore, a study determined the interaction between cell death and cellular senescence in human colorectal cancer cells induced by melatonin [170]. Melatonin treatment of 10 μM activated cell death programs and induced G_1_-phase arrest at the advanced phase of cancer cells, and induced insignificant deleterious effects on neonatal cardiomyocytes, compared with trichostatin A. Besides, a study reported a significant decrease in mRNA expression of melatonin receptor MT1 in colorectal cancer compared with the healthy adjacent mucosa tissue [171].

Melatonin has also shown synergistic effect with other anticancer agents on colorectal tumor. Combination of ursolic acid and melatonin led to an enhanced antiproliferative and pro-apoptotic activities in colon cancer cell lines SW480 and LoVo. The enhanced effects were via the cytochrome c/caspase, MMP9/COX-2, and p300/NF-κB signaling pathways [172].

#### *In vivo* studies

A study investigated the effect of melatonin on a mouse model of colitis-associated colon carcinogenesis (CACC). Melatonin decreased the progression of CACC by downregulating the process of autophagy, and alleviating the level of several inflammatory markers. Melatonin also increased expression of Nrf2 and the associated antioxidant enzymes in the colon of mice with CACC [173]. Constant light environment is related with high incidence of colon cancer, mainly through causing disorders in neuroendocrine colon system [174]. In a study, preneoplastic patterns in colon tissue from animals exposed to constant light environment (14 days; 300 lx) were analyzed, and induction of aberrant crypt foci (ACF) development was observed, characterized by increase of number of CD133^+^ and CD68^+^ cells. Increase of the proliferative process and decrease of caspase-3 protein were also observed. However, the above alterations were reversed by melatonin supplementation, through controlling the dysplastic ACF development and the preneoplastic patterns. These results pointed to that melatonin had a great potential to control the preneoplastic patterns induced by constant light [175].

Collectively, melatonin could be a new appealing therapeutic strategy for colorectal cancer, since it could regulate carcinogenesis, development, and progression of colorectal cancer. The underlying mechanisms involve multiple signaling pathways, including regulation of CaMKII, ET-1, Nrf2 signaling pathways, and induction of ACF.

### Other cancers

The anticancer effect of melatonin has also been observed in melanoma. A study revealed that melatonin combined with ER stress (induced by thapsigargin or tunicamycin) decreased cell viability of B16F10 melanoma cells, *via* the PI3K/Akt/mTOR pathway [176]. In addition, melatonin enhanced the antitumor activity of fisetin in melanoma cells, as shown by enhanced inhibition on cell viability, cell migration and clone formation, as well as the increased apoptosis. The possible mechanism might be through activation of cytochrome c-dependent apoptotic pathway and inhibition of COX-2/iNOS and NF-κB/p300 signaling pathways [177]. In another study, low melatonin concentrations (10^−9^-10^−5^ M) suppressed proliferation of B16 melanoma cells without inhibition on fibroblasts, while the high concentrations (10-4-10-2 M) inhibited the cell viability of melanoma cells, but the inhibitory activity was not as marked as that on non-tumor cell (3T3 fibroblasts). The ROS production might contribute to melatonin-induced cell viability inhibition at high melatonin concentrations [178].

Melatonin also showed anti-cancer effect on pituitary prolactin-secreting tumor. In a study, melatonin induced apoptosis of prolactinoma tumor cells *via* inducing mitochondrial dysfunction and inhibiting energy metabolism, both *in vivo* (male rats) and *in vitro* (prolactinoma cells) [179]. Elevated levels of four mitochondrial respiratory complexes activities and ATP production were observed in E-2-induced prolactin-secretory tumor cells, and melatonin repressed the activities of mitochondrial respiratory complexes and the production of ATP.

Melatonin has also shown anticancer effect on human leiomyosarcoma (LMS) [180]. Melatonin showed significant inhibitory effects on tissue-isolated human LMS xenografts, by suppressing aerobic glycolysis (Warburg effect) and tumor linoleic acid uptake and other related signaling mechanisms. Melatonin at physiological concentration also inhibited cell proliferation and cell invasion in *in vitro* cell culture studies. Another study showed that melatonin was able to induce cell death on a human alveolar rhabdomyosarcoma cell line in a dose- and time-dependent manner [181]. Furthermore, melatonin treatment at 150 and 300 μg/30 g bw for 12 consecutive days could induce a very effective oncostatic and cytotoxic activity in mice incubated with Ehrlich ascites tumor cells [182].

Furthermore, melatonin showed anti-apoptotic effects on lymphocytes and neutrophils obtained from rats injected with HL-60 leukemia cells, as shown by significantly inhibiting caspase-3 and -9 activities, and reverting the proportions of lymphocytes, neutrophils, and eosinophils to their basal values [183]. Besides, melatonin induced cell death in human malignant haematological cell lines, *via* the activation of the extrinsic pathway of apoptosis, regulated by the improvement in expression of the death receptors Fas, DR4 and DR5 and their ligands Fas L and TRAIL [184]. Moreover, a study investigated melatonin's effect on different cancer cells, including pulmonary adenocarcinoma A549 cell, chondrosarcoma sw-1353 cell, glioblastoma A172 cell, and human acute myeloid leukemia HL-60 cell. A dual effect of melatonin on intracellular redox state was found. That is, oncostatic effect of melatonin depended on its ability to induce either an antioxidant environment (leading to an antiproliferative effect in some tumors) or a prooxidant environment (leading to a cytotoxic effect in some tumors) [185].

Melatonin combined with some chemotherapeutic drugs could exert a synergistic toxic effect on A172 malignant glioma cells and brain tumor stem cells [186], *via* downregulating the expression and function of adenosine triphosphate-binding cassette transporter ABCG2/BCRP, whose overexpression in malignant glioblastomas is responsible for the multidrug resistance and tumor relapse. Besides, melatonin showed synergistic antitumor effect with vincristine and ifosfamide on SK-N-MC human Ewing sarcoma cancer cells, through potentiating extrinsic apoptosis [187].

**Figure 3:**
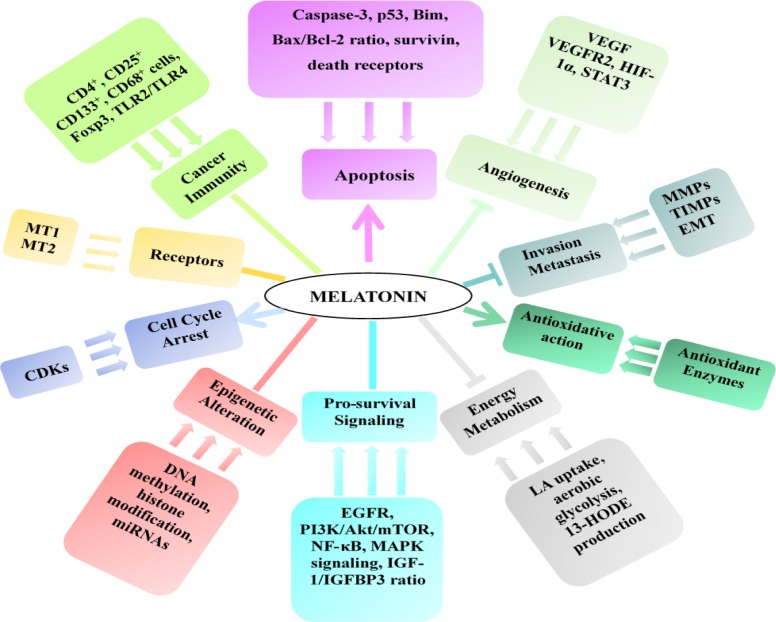
Mechanisms of the anticancer effect of melatonin →stands for promotion, - stands for regulation, and —stands for inhibition.

The anticancer effect of melatonin and possible mechanisms of action are summarized in Table 2, Table 3 and Figure 3, and the synergistic effects of melatonin with other chemotherapy or radiotherapy are summarized in Table 4.

**Table 2:** The *in vitro* and *in vivo* effects of melatonin on hormone-dependent cancers

Study Type	Subject	Dose	Main Effect	Possible Mechanisms	Ref.
**Breast Cancer**					
*in vitro*	CMT-U229 and MCF-7 cells	1 mM	inhibiting cancer cell metastasis	NA	[57]
*in vitro*	MCF-7/6, MCF-7/Her2.1, and MCF-7/CXCR4 cells	10^−9^ M	inhibiting cancer cell invasion	down-regulation of the p38 pathway and suppression of MMP-2 and -9 expression and activity	[58]
*in vitro*	10 canine mammary tumor fragments	0.5, 1, 2, 5, 10 mM	decreasing proliferation and viability and inducing apoptosis	NA	[59]
*in vitro*	MDA-MB-361 cells	1 mM	inhibiting cell proliferation and inducing apoptosis	COX-2/PGE2, p300/NF-κB, and PI3K/Akt/signaling; activation of Apaf-1/caspase-dependent apoptotic pathway	[60]
*in vitro*	MCF-7 and MDA-MB-231 cells	1 nM	anti-angiogenesis effect	NA	[56]
*in vitro*	MCF-7 cells	1 mM	anti-angiogenesis effect	NA	[62]
*in vitr*o	MDA-MB-231 cells	1 mM	anti-angiogenesis effect	NA	[63]
*in vitr*o	T47D cell	20 nM	anti-aromatase effect	acting as a selective estrogen enzyme modulators	[64]
*in vitro*	in vitro	1 nM	anti-aromatase effect	inhibiting COX expression and activity	[65]
*in vitro*	breast cancer-associated fibroblasts	10 pM, 1 nM, 10 μM	inhibiting aromatase expression and activity	inhibiting *CYP19A1* gene transcription	[66]
*in vitro*	MCF-7 cells	1 mM	decreasing aromatase activity and expression	interfering with the desmoplastic reaction via downregulating the anti-adipogenic cytokines	[67]
*in vitro*	MCF-7 cells	1 nM, 100 nM	inhibiting growth of cancer cells	inducing differential expression of miRNA and miRNA-related genes	[68, 69]
*in vitro*	MCF-7 cells	1 nM	growth inhibition effect on cancer cells	influencing DNA methylation patterns	[70]
*in vitro*	MCF-7 cells	1 μM	inhibiting cell proliferation and migration	downregulation of miR-24	[71]
*in vitro*	MCF-7 cells	1 nM, 100 nM and 10 mM	inhibiting cell proliferation	overexpressing of MT1 receptors	[72]
*in vitro*	MDA-MB-231, BT-20, SK-BR-3 cells	10^−9^ M	inhibiting proliferation of cancer cell	NA	[73]
*in vitr*o	MCF-7 and MDA-MB-231 cells	0.0001- 1 mm	controlling metastasis	modulation of ROCK-1 expression	[81]
*in vivo*	athymic nude mice	100 mg/kg bw			
*in vitro*	MDA-MB-231 cells	0.0001-1 mm	decreasing cell viability	anti-angiogenesis effect	[82]
*in vivo*	athymic nude mice	40 mg/kg bw	reducing tumor size and cell proliferation		
*in vivo*	BALB/c mice	33 mg/L in drinking water	decreasing tumor growth rate induced by LAN	global DNA methylation	[83]
*in vivo*	rats	human melatonin-rich blood	affecting cancer cell invasion	activating GSK3β	[84]
**Prostate Cancer**					
*in vit*ro	PC-3 cells	1 mM	anti-angiogenesis effect	upregulation of miRNA3195 and miRNA374b	[93]
*in vitro*	LNCaP, 22Rv1 cells	10^−8^ M	inhibiting proliferation	MT1 receptor-mediated inactivation of NF-κB	[94, 95]
*in vitro*	PC-3 cells	1 nM or 1 mM	suppressing HIF-1α accumulation	inactivating sphingosine kinase 1 pathway and scavenging free radicals	[96]
*in vitro*	multiple cancer cell lines	10 nM-2 mM	reducing proliferative potential	suppressing Sirt1 activity	[98]
*in vitro*	LNCaP cells	1 mM	sensitizing cancer cells to cytokines-induced apoptosis	causing phenotypic changes, mainly neuroendocrine differentiation	[99]
*in vitro*	LNCaP, 22Rm1, DU145 and PC3 cells	1 mM	inhibiting proliferation	resynchronizing dysregulated circadian rhythm circuitry	[100]
*in vi*vo	nude rats	amplified by day time blue light	reducing cancer metabolic, signaling, and proliferative activities	inhibiting Warburg effect	[101]
*in vivo*	mice	4 nM	inhibiting tumor growth	decreasing angiogenesis	[102]
*in vivo*	nude rats	human melatonin-rich blood (> 100 pg/mL)	dampening signal transduction, metabolic and growth activity in cancer xenografts	melatonin MT1 receptor-mediated mechanism	[103]
*in vivo*	mice	10 and 20 mg/L in tap water	inhibiting cancer tumorigenesis	suppressing Sirt1 activity	[98]
**Ovarian Cancer**					
*in vitro*	OVCAR-429 and PA-1 cells	400, 600, and 800 μM	inhibition on tumor growth	delay of G_1_/S via down-regulation of CDK2 and 4	[106]
*in vivo*	rats	200 μg/100 g	reducing tumor masses and incidence of adenocarcinomas	NA	[108]
*in vivo*	rats	200 μg/100 g	reducing tumor masses and inducing apoptosis	upregulation of p53, BAX, and cleaved caspase-3, and enhancement of DNA fragmentation	[109]
*in vivo*	rats	200 μg/100 g	reducing tumor volume	attenuating the TLR4-induced MyD88- and TRIF-dependent signaling pathways	[110]
*in vivo*	rats	200 μg/100 g	reducing tumor masses	attenuating Her-2, p38 MAPK, p-AKT, and mTOR Levels	[112]
**Cervical Cancer**					
*in vivo*	nude rats	500 pM	inhibiting tumor growth	inhibiting aerobic glycolysis and fatty acid metabolic signaling	[115]
*in viv*o	nude rats	500 pM	suppressing tumor metabolism and proliferation	inhibition of linoleic acid transport and 13-HODE production	[116]

NA stands for not available.

**Table 3:** The *in vitro* and *in vivo* activities of melatonin on hormone-independent cancers

Study Type	Subjects	Dose	Main Effect	Possible Mechanisms	Ref.
**Oral Cancer**					
*in vitro*	HSC-3, OECM-1 cells	0.5, 1 mM	anti-metastatic effect	attenuation of MMP-9 expression and activity mediated by decreasing histone acetylation	[121]
*in vitro*	SCC9 cells	1 mM	decreasing cell viabilities	inhibiting the expression of *HIF-1α*, *VEGF*, and *ROCK-1* genes	[122]
**Liver Cancer**					
*in vitro*	HepG2 cells	1 mM	modulating motility and invasiveness	upregulation of TIMP-1 and attenuation of MMP-9 via NF-κB signaling pathway inhibition	[126]
*in vitro*	HepG2 cells	1 mM	anti-angiogenesis activity	interfering with transcriptional activation of VEGF, via Hif1α and STAT3	[127]
*in vitro*	HepG2, SMMC-7721 cells	10^−9^, 10^−7^, 10^−5^, 10^−3^ M	overcoming apoptosis resistance	suppressing survivin and XIAP via the COX-2/PI3K/AKT pathway	[128]
*in vitro*	HepG2 cells	50 to 2000 μM	pro-apoptotic effect	regulation of Bim by FoxO3a	[129]
*in vitro*	HepG2 cells	1000 and 2500 μM	inhibiting proliferation	modulation of MT1 membrane receptor and cAMP and ERK activation	[130]
*in vivo*	HepG2 cells	10^−9^, 10^−7^, 10^−5^, 10^−3^ M	sensitizing cancer cells to ER stress-induced apoptosis	downregulating the COX-2 expression and the Bcl-2/Bax ratio, elevating level of CHOP	[131]
*in vivo*	mice	0.5 mg/kg	reversing the circadian clock disturbed by hepatocarcinogenesis	NA	[132]
*in vivo*	rats	1 mg/kg	attenuating hepatocarcinogenes	activating ER stress	[133]
**Renal Cancer**					
*in vitro*	Caki-1 and Achn cells	0.5-2 mM	anti-metastatic effect	suppressing Akt-MAPKs pathway and NF-κB DNA-binding activity	[135]
*in vitro*	Caki cells	0.1, 0.5, or 1 mM	inducing apoptosis	upregulation of Bim expression	[136]
*in vitro*	Caki cells	1 mM	enhancing apoptosis induced by thapsigargin	ROS-mediated upregulation of CCAAT-enhancer-binding protein homologous protein	[137]
*in vitro*	Caki cells	1 mM	enhancing apoptosis induced by kahweol	inducing upregulation of p53-upregulated modulator of apoptosis	[138]
**Lung Cancer**					
*in vitro*	A549 cells	0.1, 0.5, 0.75, 1.0, 2.5, 5.0 mM	suppressing cell migration and viability	JNK/MAPK Pathway	[143]
**Gastric Cancer**					
*in vitro*	SGC-7901 cells	0.01, 0.1, 1, 3 mM	inhibited HIF-1α accumulation and endogenous VEGF generation	inhibition of melatonin nuclear receptor RZR/RORγ	[147]
*in vitro*	AGS cells	0.25, 0.5, 1, 2, 4 mM	inhibiting cell viability, clone formation, cell migration and invasion, and inducing apoptosis	activation of JNK and P38 MAPK and the suppression of NF-κB	[149]
*in vitro*	SGC-7901 cells	10^−9^-10^−3^ M	inhibiting cell viability, clone formation, cell migration, and promoting apoptosis	NA	[150]
*in vitro*	murine MFC cell	4 mM	inhibiting cancer growth	increase of p21 and Bax and decrease of Bcl-2 mediated by membranous melatonin receptors	[151]
*in vitro*	SGC-7901 cells	10^−4^ M	inducing cell differentiation	upregulation of endocan, downregulation of alkaline phosphatase and lactate dehydrogenase activities	[153]
*in vitro*	murine MFC cell	2, 4, 6, 8, 10 mM	promoting apoptosis and inducing cell cycle arrest at the G_2_/M phase	downregulation of CD4^+^CD25^+^ cells and its Forkhead box p3 expression	[152]
*in vivo*	mice	25, 50, 100 mg/kg	reducing tumor tissue		
*in vitro*	SGC-7901 cells	3 mM	inhibiting angiogenesis	suppressing RZR/RORγ, SENP1, HIF-1α, and VEGF	[148]
*in vivo*	nude mice	NA	reducing tumor volume and weight and inhibiting proliferation and angiogenesis		
*in vivo*	BALB/c mice	5 mg/kg/twice/week	impeding tumor growth and peritoneal dissemination	inducing ER stress and inhibiting EMT	[154]
**Pancreatic Cancer**					
*in vitro*	PANC-1 cells	1 mM	inhibiting cell proliferation and angiogenesis	decreasing VEGF	[158]
*in vitro*	AR42J cells	1 mM	reducing cell viability	inducing changes of mitochondrial activity and activating caspase-3	[159]
**Colorectal Cancer**					
*in vitro*	HCT 116 cells	10^−6^ M	decreasing cancer cellular viability	increasing ROS level	[164]
*in vitro*	HT-29 cells	10^−6^-10^−2^ M	antiproliferative effect	the antioxidative and anti-inflammatory actions	[165]
*in vitr*o	LoVo cells	10^−4^, 10^−3^, 10^−2^, 10^−1^, 1, 2 mM	suppressing cell proliferation and inducing apoptosis	HDAC4 nuclear import mediated by CaMKII inactivation	[166]
*in vitro*	Caco-2 and T84 cells	0.1, 0.25, 0.5, 1 mM	inhibiting tumor growth and progression	repressing the activation of ET-1	[168]
*in vitro*	Caco-2 cells	1.56, 0.78 μg/mL	inducing morphological changes in cancer cell	generation of ROS	[169]
*in vitro*	HCT116 cells	10 μM	activating cell death programs early	inducing G_1_-phase arrest	[170]
*in vivo*	mice	1mg/kg bw	inhibiting the progression of colitis-associated colon carcinogenesis	preventing the process of autophagy, alleviating the level of several inflammatory markers, and modulating Nrf2 signaling pathway	[173]
*in vivo*	rats	10 mg/kg	controlling the preneoplastic patterns	controlling the dysplastic ACF development	[175]
**Melanoma**					
*in vitro*	B16 cells	10^−4^-10^−2^ M	reducing cell viability	promoting ROS production	[178]
Prolactinoma					
*in vitro*	prolactinoma cells	10^−5^-10^−3^ M	inducing apoptosis	repressing activities of mitochondrial respiratory complexes and production of ATP	[179]
*in viv*o	rats	0.25 or 0.50 mg/d			
**Leiomyosarcoma**					
*in vitro*	SK-LMS-1 cells	100 nM-1 pM	repressing cell proliferation and cell invasion	suppression of aerobic glycolysis and survival signaling	[180]
*in vivo*	nude rats	human melatonin-rich blood	inhibiting tumor proliferative activity		
**Alveolar Rhabdomyosarcoma**					
*in vitro*	RH30 cells	1, 2 mM	inducing cell death	NA	[181]
**Ehrlich Ascites Carcinoma**					
*in vivo*	mice	5, 10 mg/kg	oncostatic and cytotoxic activity	NA	[182]
Leukaemia					
*in vivo*	Rats injected with HL-60 cells	20 μg/mL in tap water	anti-apoptotic effects on lymphocytes and neutrophils	inhibiting caspase-3 and -9 activity	[183]
*in vitro*	human malignant haematological cell	1 mM	inducing cell death	activation of the extrinsic pathway of apoptosis	[184]

NA stand for not available.

**Table 4:** The synergetic effect of melatonin with other anticancer drugs or radiotherapy

Cancer Category	Study Type	Treatment	Main Effect	Ref.
breast cancer	*in vitro*	melatonin (0.3 mM) + doxorubicin (0.5 or 1μM)	inducing apoptosis and cell death by activating TRPV1	[74]
breast cancer	*in vitro*	melatonin (0.5-5 μM) + arsenic trioxide (0.5-5 μM)	enhancing the apoptotic cell death by generation of ROS, upregulation of Redd1 expression, and activation of the p38/JNK pathways	[75]
breast cancer	*in vitro*	melatonin (3 mM) + puromycin (1 μM)	inhibiting cancer cell viability by inhibiting 45S pre-rRNA and XPO1 and downregulating IPO7, procaspase 3 and Bcl-xL	[76]
breast cancer	*in vitro*	melatonin (100 μM) + all-trans retinoic acid (1 μM) + Somatostatin (1 μM)	enhancing the growth inhibitory effect	[77]
breast cancer	*in vitro*	melatonin (1 nM) + vitamin D_3_ (1 nM)	inhibiting cell proliferation through a TGFβ-1-dependent mechanism	[78]
breast cancer	*in vitro*	melatonin (1 nM-1 mM) + ionizing radiation (0-12 Gy)	sensitizing cancer cells to radiation by downregulating proteins involved in double-strand DNA break repair	[79]
breast cancer	*in vitro*	melatonin (1 nM-1 mM) + radiation (8 Gy)	enhancing radiosensitivity of cancer cell by modulation on p53	[80]
breast cancer	*in vivo*	melatonin (20 mg/L) + *L. plantarum* LS/07 (8.4 ´ 10^8^ c.f.u.) + inulin (20 g/kg)	augmenting the pro-differentiating, antiproliferative activities via enhancing immunomodulatory action	[85]
breast cancer	*in vivo*	melatonin (10 mg/kg) + *P. acnes* (1 ´ 10^6^ cells)	reducing angiogenesis, inhibiting metastasis, inducing apoptosis by stimulating strong Th1-type cytokine antitumor immune response	[86]
breast cancer	*in vivo*	melatonin + adriamycin	sensitizing tumor to adriamycin	[87]
breast cancer	*in vivo*	melatonin (20 g/mL) + pravastatin (100 mg/kg)	enhancing the anti-tumor effect of pravastatin	[88]
breast cancer	*in vivo*	melatonin (0.1 μg/mL) + doxorubicin (6 mg/kg)	inhibiting tumor metabolism, reestablishing the sensitivity of breast tumors to doxorubicin and driving tumor regression by inhibition on circadian-regulated kinase	[89]
breast cancer	*in vivo*	melatonin (0.1 μg/mL) + tamoxifen (80 mg/kg)	inhibiting tumor metabolism, reestablishing the sensitivity of breast tumors to tamoxifen and driving tumor regression by inhibition on circadian-regulated kinase	[90]
ovarian cancer	*in vitro*	melatonin (0-2 mM) + cisplatin (80 μM)	enhancing cisplatin-induced apoptosis by inactivation of ERK/p90RSK/HSP27 cascade	[107]
cervical cancer	*in vitro*	melatonin (1 mM) + cisplatin (20 μM)	reducing cell viability and enhancing cytotoxicity of cisplatin by ROS overproduction and enlarged DNA fragmentation	[114]
endometrial cancer	*in vivo*	melatonin (25 μg/mL) + estrogen	decreasing endometrial proliferation and preventing the appearance of cellular atypia	[119]
lung cancer	*in vitro*	melatonin (0.1 mM) + UV irradiation	enhancing apoptosis induced by UV irradiation	[142]
lung cancer	*in vitro*	melatonin (1 mM) + berberine (100 μM)	sensitizing cancer cells to berberine via activation of caspase/Cyto C and inhibition of AP-2β/hTERT, NF-κB/COX-2 and Akt/ERK signaling pathways	[140]
lung cancer	*in vitro*	melatonin (1 mM) + gefitinib (1μM)	downregulating EGFR phosphorylation and inducing apoptosis by sensitizing TKI-resistant cell to gefitinib	[144]
pancreatic cancer	*in vitro*	melatonin (1.5 mM) + gemcitabine (20 mM)	inducing apoptosis and necrosis by modulation of Bcl-2/Bax balance	[160]
	*in viv*o			
pancreatic cancer	*in vitro*	melatonin (1 mM) + 5-fluorouracil (1 mM) or cisplatin (20 μM) or doxorubicin (1μM)	enhancing cytotoxicity and apoptosis by increasing intracellular ROS production and enhancing mitochondrial membrane depolarization	[161]
pancreatic cancer	*in vivo*	melatonin (20 μg/mL) + capecitabine (50 mg/d)	improving antitumor activity by decreasing lipoperoxide levels and increasing antioxidant activity	[162]
colorectal cancer	*in vitr*o	melatonin (1.0 mM)) + ursolic acid (20 μM)	enhancing antiproliferative and pro-apoptotic activities by regulation of cytochrome c/caspase, MMP9/COX-2, and p300/NF-κB signaling pathways	[172]
melanoma	*in vitro*	melatonin (0.1-1.0 mM) + thapsigargin (1 μM) or tunicamycin (5 μg/mL)	decreasing cell viability via regulation of the PI3K/Akt/mTOR pathway	[176]
melanoma	*in vitro*	melatonin (0.1-1.0 mM) + fisetin (20μM)	enhancing the antitumor activity by inhibition of COX-2/iNOS and NF-κB/p300 signaling pathways	[177]
glioblastomas	*in vitro*	melatonin (1 mM) + temozolomide (0–2 mM) or doxorubicin (0–50 mM) or mitoxantrone (0–50 mM)	inducing a synergistic toxic effect on cancer cell by increasing methylation of the ABCG2/BCRP promoter	[186]
Ewing sarcoma	*in vitro*	melatonin (1 mM) + vincristine (5 nM) or ifosfamide (0.5 mM)	exerting synergistic antitumor effect by potentiating extrinsic apoptotic pathway	[187]

## CLINICAL TRIALS

In clinical trials concerning melatonin's anticancer effect, melatonin was mainly used as an adjuvant therapy with other chemotherapeutic drugs. Several clinical studies suggested that melatonin could enhance the therapeutic efficacy and reduce the toxicities of other anticancer drugs, as shown by increased partial response, induced tumor regression, higher survival rate and relieved symptoms of side effects. According to a clinical trial, treatment of low-dose subcutaneous interleukin-2 (3 million IU/day for 6 days/week for 4 weeks) plus melatonin (40 mg/day orally) significantly increased 1 year survival rate of patients with metastatic colorectal cancer, compared with supportive care alone (9/25 *vs*. 3/25, *p* < 0.05) [188]. In a phase-II study including 14 patients with metastatic breast cancer, an oral 20 mg/day of melatonin starting 7 days before tamoxifen therapy achieved a partial response in 4/14 (28.5%) patients, caused a relief of anxiety in most patients, and did not enhance the toxicity of tamoxifen. Besides, serum levels of IGF-1 were decreased by the combination therapy [189]. Another clinical trial also reported that melatonin could enhance the efficacy of chemotherapy and reduce the toxicity in patients with metastatic solid tumor [190]. Besides, a clinical study evaluated the effect of a concomitant administration of melatonin (20 mg/day orally in the evening) on metastatic NSCL cancer patients receiving a chemotherapeutic regimen consisting of cisplatin and etoposide. Patients receiving concomitant melatonin showed higher overall tumor regression rate and 5-year survival, with a better tolerance to chemotherapy [191]. Similarly, concomitant melatonin with irinotecan achieved a higher percent of disease-control on metastatic colorectal cancer patients than irinotecan alone, because partial response and stable disease were obtained by more patients [192].

Besides, several studies reported that melatonin might be able to improve the sleep and quality of life in patients with breast cancer. In a prospective phase II trial, bedtime melatonin was associated with a significant improvement in objective sleep quality, subjective sleep, sleep fragmentation and quantity, fatigue severity, global quality of life, and social and cognitive functioning scales [193]. Besides, a double-blind, placebo-controlled, randomized clinical trial pointed out that the risk of developing depressive symptoms in subjects who received 6 mg oral melatonin was significantly lower than that of subjects who took placebo [194]. Furthermore, the secondary outcomes of this trial reported that 6 mg oral melatonin administration approximately 1 hour before bedtime led to significantly improved sleep efficiency and reduced wake after sleep onset for the 2-week postoperative period [195]. Another randomized, placebo-controlled trial claimed that compared to subjects on placebo, subjects who received melatonin reported significantly increases in subjective sleep quality as measured by the Pittsburgh Sleep Quality Index (PSQI) [196]. Another study showed that melatonin combined with somatostatin, retinoids, vitamin D_3_ and low dose of cyclophosphamide preformed a positive action in terms of efficacy and survival of breast cancer in human [197]. However, a double-blind, placebo-controlled study reported that short-term melatonin treatment did not produce any significant influence on the estradiol and IGF-1/IGBBP-3 levels in women with a prior history of stages 0-III breast cancer [198]. Furthermore, another double-blind placebo-controlled crossover trial which included 72 patients suggested that 20 mg oral melatonin administration was not able to improve fatigue or other symptoms in patients with advanced cancer [199].

Collectively, in clinical trials, melatonin showed the ability to enhance the therapeutic effect of various anticancer drugs. Meanwhile, melatonin might help improving the sleep and life quality of cancer patients.

## CONCLUSIONS AND PROSPECTS

The effects of melatonin on cancers have been widely studied, with a focus on hormone-dependent cancers. Epidemiological studies concerning the association between body circadian melatonin levels and cancer incidence led to controversial conclusions, which were either significant association or no association at all. Numerous experimental studies have indicated an oncostatic role of melatonin in various cancers, such as breast, ovarian, prostate, oral, gastric, and colorectal cancers. The underlying mechanisms include several molecular pathways, which are associated with antioxidant activity, modulation of melatonin receptors MT1 and MT2, regulation of apoptosis, pro-survival signaling and tumor metabolism, inhibition of angiogenesis, invasion and metastasis, and induction of epigenetic alteration. Melatonin also showed the potential to be utilized as adjuvant of cancer therapies, through reinforcing the therapeutic effects and reducing the side effects of chemotherapies or radiation. In clinical trials, melatonin showed the ability to enhance the therapeutic effect of various anticancer drugs, and might help improving the sleep and life quality of cancer patients. Overall, the impressive efficacy and safety of melatonin support it as a promising agent for the prevention and treatment of cancers.

In the future, several aspects about melatonin's anticancer action should be further investigated. For epidemiological studies, the main problem is the inconsistence of results. This might be because different kinds of sample, sample collection time and assessment methods of melatonin were used. Therefore, different assessment methods of melatonin need to be compared, and the most reliable one should be adopted in future studies. Besides, the effects of different kinds of sample (such as urine, plasma or serum) on the results need to be studied, and the same type of sample should be used. Furthermore, the most appropriate sample collection time should be determined because the melatonin concentration in human body changes with circadian rhythm. For experimental studies, it should be noted that melatonin regulates the physiology and molecular biology of cells through a variety of mechanisms, and cancer is a heterogeneous disease. Therefore, the anticancer efficacy of melatonin was not limited to the aforementioned mechanisms of action. For incidence, autophagy is a prominent feature of programmed cell death, and studies of melatonin's effect on autophagy in cancer cells are very few. Besides, beneficial effects of melatonin against mitochondrial dysfunction in different pathologies have also been described in literature. Thus, there is a possibility that melatonin's oncostatic effect is linked to mitophagy. However, the related researches in this topic are insufficient. Thus, future research might encompass melatonin's effect on autophagy and mitophagy, and other molecular mechanisms involved in its anticancer action. For clinical trials, melatonin's enhancing effect on more anticancer drugs should be further assessed. In addition, its direct effect on patients with manifest cancer should be studied by exogenous melatonin administration to find its oncostatic effects on some cancers and provide information on dosage and long-term safety of melatonin. Moreover, mechanisms of action should be investigated further.

## Abbreviations

The following abbreviations are used in this manuscript:

ACF: aberrant crypt foci
ALT: alanine amino transferase
aMT6s: 6-sulfatoxymelatonin
Apaf-1: apoptotic protease activating factor 1
AR: androgen receptor
AST: aspartate aminotransferase
Bax: Bcl-2 associated X protein
Bcl-2: B-cell lymphoma-2
Bcl-xL: B-cell lymphoma-xL
Bim: Bcl-2-interacting mediator
CACC: colitis-associated colon carcinogenesis
cAMP: cyclic adenosine monophosphate
CaMKII: Ca^2+^/calmodulin-dependent protein kinase IIα
CCAR2: cell cycle and apoptosis regulator 2
CHOP: C/EBP homologous protein
COX: cyclooxygenase
DR4: death receptor 4
DR5: death receptor 5
EMT: epithelial-mesenchymal transition
EGR: early growth response
EGFR: epidermal growth factor receptor
ER: endoplasmic reticulum
ERK: extracellular regulated protein kinase
ET-1: endothelin-1
Fas: factor associated suicide
Foxp3: Forkhead box p3
GPC3: glypican-3
GPCR: G-protein-coupled receptor
GSK3β: glycogen synthase kinase 3β
HCC: hepatocellular carcinoma
HDAC4: histone deacetylase 4
Her-2: human epidermal growth factor receptor2
IAPs: inhibitor of apoptosis proteins
IGF-1: insulin-like growth factors
IGFBP-3: insulin-like growth factor-binding protein 3
HIF: hypoxia-inducible factor
HSP: heat shock protein
IPO7: importin-7
JNK: c-Jun N-terminal kinase
LAN: light at night
LMS: leiomyosarcoma
MAPK: mitogen-activated protein kinase
MDA: malondialdehyde
MFC: murine foregastric carcinoma
miRNA: microRNA
mTOR: mechanistic target of rapamycin
MMP: matrix metalloproteinase
MyD88: myeloid differentiation factor 88
NF-κB: nuclear factor-κB
NSCLC: non-small cell lung cancer
P53: tumor protein 53
PC-3: prostate cancer cells
PCNA: proliferating cell nuclear antigen
Per2: Period 2
PI3K: phosphatidylinositol 3-kinase
PKA: protein kinase A
PKB: protein kinase B
PKC: protein kinase C
PGE: prostaglandin E
REDD1: regulated in DNA damage and development 1
RCC: renal cell carcinoma
ROCK: Rho-associated protein kinase
ROS: reactive oxygen species
Sirt1: sirtuin 1
SCC: squamous cell lung carcinoma
SENP1: sentrin-specific protease 1
STAT3: signal transducers and activators of transcription 3
TGF: transforming growth factor
TIMP: tissue inhibitor of metalloproteinases
TKIs: tyrosine kinase inhibitors
TLR: toll-like receptors
Tregs: regulatory cells
TRIF: TIR-domain-containing adapter-inducing interferon-β
TNF: tumor necrosis factor
TRAIL: TNF related apoptosis inducing ligand
TRPV1: transient receptor potential vanilloid 1
VEGF: vascular endothelial growth factor
XIAP: X-linked inhibitor of apoptosis
XPO1: exportin 1.
